# Oceanographic Conditions Limit the Spread of a Marine Invader along Southern African Shores

**DOI:** 10.1371/journal.pone.0128124

**Published:** 2015-06-26

**Authors:** Jorge Assis, Mirta Zupan, Katy R. Nicastro, Gerardo I. Zardi, Christopher D. McQuaid, Ester A. Serrão

**Affiliations:** 1 Center of Marine Sciences, University of Algarve, Faro, Portugal; 2 Department of Zoology and Entomology, Rhodes University, Grahamstown, South Africa; Evolutionary Biology Centre (EBC), Uppsala University, SWEDEN

## Abstract

Invasive species can affect the function and structure of natural ecological communities, hence understanding and predicting their potential for spreading is a major ecological challenge. Once established in a new region, the spread of invasive species is largely controlled by their dispersal capacity, local environmental conditions and species interactions. The mussel *Mytilus galloprovincialis* is native to the Mediterranean and is the most successful marine invader in southern Africa. Its distribution there has expanded rapidly and extensively since the 1970s, however, over the last decade its spread has ceased. In this study, we coupled broad scale field surveys, Ecological Niche Modelling (ENM) and Lagrangian Particle Simulations (LPS) to assess the current invaded distribution of *M*. *galloprovincialis* in southern Africa and to evaluate what prevents further spread of this species. Results showed that all environmentally suitable habitats in southern Africa have been occupied by the species. This includes rocky shores between Rocky Point in Namibia and East London in South Africa (approx. 2800 km) and these limits coincide with the steep transitions between cool-temperate and subtropical-warmer climates, on both west and southeast African coasts. On the west coast, simulations of drifting larvae almost entirely followed the northward and offshore direction of the Benguela current, creating a clear dispersal barrier by advecting larvae away from the coast. On the southeast coast, nearshore currents give larvae the potential to move eastwards, against the prevalent Agulhas current and beyond the present distributional limit, however environmental conditions prevent the establishment of the species. The transition between the cooler and warmer water regimes is therefore the main factor limiting the northern spread on the southeast coast; however, biotic interactions with native fauna may also play an important role.

## Introduction

Invasive species represent one of the main threats to global biodiversity, affecting the function and structure of natural ecological communities and causing annual economic losses amounting to billions of dollars worldwide [1–4]. Since the publication of Charles Elton’s book “The ecology of invasions by animals and plants” in 1958 [5], biological invasions have gained exponentially growing attention [6]. The realization that most successful invasions are irreversible [7] has motivated ecologists to attempt to identify the environmental factors influencing the spread of invaders in new ecosystems and to quantify and forecast the success of extant invasive processes (e.g., [8–14]). While most of these studies assume that the spread of new colonizers is primarily controlled by the potential for invasive species to disperse and the availability of suitable habitats in the new environments, the use of fragmented approaches focusing on only one of these possibilities may have precluded important conclusions regarding invasive processes.

Many marine species have a planktonic dispersal phase and the distance that propagules can travel is often positively correlated to their Pelagic Larval Duration (PLD; but see [15]). As a result, many studies have adapted Lagrangian Particle Simulations (LPS; see [16–17] for a review) to assess the potential for invasive species to disperse from neighbouring regions where establishment has already taken place [18–21]. These simulations allow the tracking of virtual particles advected by ocean current fields (e.g., [22]), and their realism can be increased by including important biological parameters such as larval behaviour or the PLD of drifting particles (e.g., [23–24]). However, the potential dispersal distance, as measured by LPS, and the realized one, often differ when behavioural strategies, local hydrodynamic conditions and most importantly, habitat suitability are not taken into account [25]. When propagules arrive at new environments, the establishment success and abundance of invasive species are dependent on the availability of suitable habitats, which is largely determined by local abiotic conditions such as climate [9,11] and biotic interaction with the indigenous biota [26,27]. Ecological Niche Models (ENM; see [28] for a review) can be used alongside LPS to predict suitable habitats for putative invasions in non-native regions. However, a major challenge arises in using ENM in the invasive context because the fundamental niche of a species may diverge between native and invasive ranges due to local selection in the face of novel environmental conditions [29–30]. This results in the violation of one of the fundamental assumptions of ENM due to an asymmetric transference of model fitting between environmental spaces [31]. Thus, a crucial step in applying ENM for invasive species is to test the agreement between the environmental spaces used by both the native and the invasive populations [32].

Among marine animals, one of the most successful invasive species is the mussel *Mytilus galloprovincialis*. This species is native to the Mediterranean and has an antitropical distribution [33], being recorded to date on the coasts of all continents except Antarctica [34]. It possesses many features that make it a successful invasive species, including high fecundity [26], fast growth, competitive abilities [34–36] and ease of transport of larvae and adults in ballast waters [37]. *M*. *galloprovincialis* arrived in South Africa in the 1970’s, probably with shipping from a Mediterranean source population [38]. It rapidly colonized the west and south coastlines of southern Africa, but its spread appears to have ceased over the last decade [39–40]. The arrival of this invader had significant ecological impacts (reviewed by [34]). On the west coast of South Africa, it has replaced the indigenous mussels *Aulacomya ater* and *Choromytilus meridionalis* as the dominant species. On sheltered shores, the limpets *Scutellastra granularis* and *S*. *argenvillei* have also been negatively affected. Despite several negative effects, the presence of *M*. *galloprovincialis* in the South African intertidal has moved the centre of distribution of intertidal mussel beds higher on the shore thus increasing the overall mussel biomass. This has important positive effects for intertidal predators such as the African black oystercatcher *Haematopus moquini*. On the west coast, this near-threatened species has shifted its diet and now feeds primarily on the invasive mussel [41]. Critically, the economic impact of *M*. *galloprovincialis* has also been significant as the South African mussel culture industry is mostly based on this invasive species [42].

In this study, we combine extensive field surveys, ENM using climatic variables and LPS approaches to assess the current invasive distribution of *M*. *galloprovincialis* in southern Africa and evaluate whether dispersal and/or environmental conditions limit the further spread of this marine invader in southern Africa. Specifically, we: (1) assessed the potential and realized niche of *M*. *galloprovincialis* in the invaded range, (2) inferred whether there might have been niche adaptation (divergence) between native and recent establishments and (3) tested the null hypothesis of no potential for larval dispersal beyond the already established non-native ranges due to major oceanographic discontinuities promoted by ocean currents.

## Materials and Methods

### Ethics Statement

All field surveys did not involve disruptive sampling and thus no specific permits were required with the exception of the South African surveys that were performed under permit number RES2014/12 issued by the Department of Agriculture Forestry and Fisheries to the Department of Zoology and Entomology at Rhodes University.

### Data on species records and climate

To have georeferenced occurrences of *Mytilus galloprovincialis* in southern Africa, we combined two types of datasets: wide-ranging field surveys and existing literature. The field surveys were carried out during low spring tides between 2010 and 2013 on rocky intertidal shores along the southern African coastline from Mowe (9.37°S, 12.70°E), in Namibia, to Ponta do Ouro (26.85°S, 32.89°E), in Mozambique. Most of the locations were visited twice, covering winter and summer months; two observers performed searches lasting approximately 60 min across all microhabitats present. Because invasive ENMs benefit from records that include species native ranges [43], we further expanded the field surveys to Northern Africa (Western Sahara, Morocco and Tunisia) and southern Europe. These were performed between 2008 and 2013 following the same procedure as for the surveys in the invasive range. The literature search covering native records also included the GBif database. Since *M*. *galloprovincialis* can hybridise with the congeneric *M*. *edulis* [44] and *M*. *trossulus* [44–45] in northern Europe, these regions were excluded. While the modelling approach used (Boosted Regression Trees; see Ecological Niche Modelling section) can account for some degree of spatial autocorrelation [46], completely neglecting the effect of spatial dependence among occurrence records may lead to poorly estimated regression coefficients and to the selection of unimportant environmental predictors [47]. To reduce spatial autocorrelation in our models, all occurrence records were gridded (0.08° x 0.08° resolution) and duplicate entries were considered only once (e.g., [48]).

Environmental predictors commonly used for intertidal species (e.g., [49–52]) were produced by determining the long-term averages (2000–2010) summarizing the annual extremes in sea surface temperature, air temperature, sea surface salinity and cloud cover. The raw environmental information was gathered from the Operational Sea Surface Temperature and Sea Ice Analysis (OSTIA) [53], the World Ocean Database 2013 [54] and the European Centre for Medium-Range Weather Forecasts (ECMWF) [55]. All predictors were gridded with bilinear interpolation to match the resolution of the distribution data.

### Ecological Niche Modelling

The niche modelling implemented in our study was performed following previous studies with high predictive performance when modelling intertidal species [52,56]. In the framework used, we identified a set of models with higher potential for spatial transferability (i.e., the ability to predict distributions in regions outside the domain of known records of occurrence; [57]) among numerous candidate models. We adopted the machine-learning method Boosted Regression Trees (BRT; [58]) because of its ability to model non-linear relationships and detect complex interactions among predictors.

Because *M*. *galloprovincialis* may be absent from some regions of southern Africa due to factors other than simple niche availability (e.g., dispersal constraints), the models were performed with pseudo-absences rather than the absences collated from surveys. The selection of this information was performed following the recommendations of Barbet-Massin et al. [59] for Boosted Regression Trees, by using the same number of pseudo-absences as the presence records, randomly selected from regions outside the species’ ecological domain (throughout both native and non-native ranges). In this step, we computed a habitat suitability map using Mahalanobis distance, relating all presence records with the normalized environmental predictors (see [60] for details). However, the threshold allowing one to extract pseudo-absences from the suitability gradient has a direct effect on both predicted areas and accuracy scores, with low thresholds for instance (e.g., 0.1), elevating accuracy scores while over-predicting distributions [61]. We adopted a threshold of 0.2, which allows a good balance between accuracy scores and predicted distributions close to realised ranges (see [61] for details).

A cross-validation framework was implemented by randomly dividing both presence and pseudo-absence records into two independent datasets, one set with 70% of the records for model training and another with 30% to test the model’s accuracy. Numerous models were trained using all possible combinations of environmental predictors with no signs of strong correlation (Spearman’s R < |0.7|). Fitting each BRT model was a process dependent on the learning rate, tree complexity, number of trees and bag fraction (see [58,62] for details). The optimal values for these parameters were determined by 10-fold cross-validation performed in the training dataset, using deviance reduction as an estimate of success (e.g., [62]). Each model was fitted using a range of learning rates (0.1, 0.05, 0.01, 0.005, 0.001) and tree complexities (from 1 up to the number of predictors in a given subset). The optimal number of trees was identified by varying values of trees from 100 to 10000 (step 50) and ensuring that at least 1000 trees were fitted [58,62]. A default value of 0.5 for bag fraction was used because it consistently results in optimal binomial responses [62]. Careful identification of the optimal parameters allowed an increased level of generality, while avoiding the problems of over fitting when predicting distributions. The accuracy of each combination of predictors was then verified using True Skill Statistics (TSS) [63], by comparing the test dataset with a reclassified predictive map that maximizes the ability to detect true absences and presences (specificity and sensitivity, respectively). Using these statistics, accuracy scores higher than 0.8 were interpreted as an excellent model, from 0.8 to 0.6 a good model, from 0.6 to 0.2 a fair to poor model, and values lower than 0.2 a model with no predictive ability.

The cross-validation framework was conducted 30 times and, for each one, the pseudo-absences were randomized along with the training and testing datasets. The contribution of the environmental predictors to the responses of models was determined by the mean accuracy when a predictor was modelled alone (univariate effect) and the mean gain in accuracy when added to a model with other predictors. To identify the combination of predictors with the highest potential for transferability we ran independent tests with the null hypothesis of no differences between the mean TSS. This was performed by sorting the combinations of predictors by decreasing accuracy scores and successively adding them to non-parametric Kruskal-Wallis rank tests until the level of significance was reached (alpha = 0.05).

The final predictive map was produced with the full set of distribution records. However, because several models could be equally transferrable (no differences in their mean accuracy), we merged (with a median function) the predictive surfaces resulting from those models trained with the highest transferrable combinations of predictors (i.e. ensemble modelling), an approach that reduces the inherent uncertainty of predictions [52,64]. The ensemble was then reclassified with a threshold maximizing the sum of sensitivity and sensibility, so the output would reflect predicted presences and absences rather than the probability of occurrence. The standard deviation of the ensemble was also computed as a measure of uncertainty. Finally, to compare the available niche with the current realized distribution of *M*. *galloprovincialis*, we determined the sensitivity and specificity of the ensemble against our field observations (both presences and absences). This approach allowed verification of the proportion of surveyed sites with suitable environmental conditions already invaded by the species.

### Niche divergence between ranges

Principal Component Analysis (PCA) was used to infer niche divergence between native and non-native ranges (e.g., [65]). All presence records were used to extract the values from the environmental predictors included in the ensemble modelling (i.e. those making the greatest contribution to the niche of this species). In this step, the predictors were scaled to have a mean of zero and a standard deviation of one. The first two PCA components were plotted and their raw values used to test the significance of niche evolution (i.e., divergence) by permutational multivariate ANOVA (PERMANOVA; [66]) performed on the Euclidian similarity matrix under 9999 randomizations, using ranges as independent variables. In this analysis, the rejection of the null hypothesis suggests niche evolution due to differences in the relative position of environmental data in the multivariate space, in their degree of dispersion, or both [67]. To disentangle the possible causes for niche divergence, we performed a permutational analysis of multivariate dispersion (PERMDISP; [67]).

### Lagrangian Particle Simulations

At large spatial scales, the larvae of *M*. *galloprovincialis* are dispersed as passive particles and their dispersal patterns and range of dispersal can be linked closely to hydrogeographic data [68]. Thus, the probability of *M*. *galloprovincialis* dispersing beyond the already established range in southern Africa was inferred by individual-based Lagrangian Particle Simulations (LPS). Data for currents were derived from the Hybrid Coordinate Ocean Model (HYCOM), a daily high-resolution product forced by wind speed, wind stress, heat flux and precipitation [69]. This model can resolve oceanic eddies, meandering currents, filaments and fronts [69], important mesoscale processes required to simulate accurately dispersing larvae (e.g., [70]). The LPS covered two separate coasts where the northern range limits are presently defined: (1) the west coast from Capulo (Angola; 8.00° S) to Walvis Bay (Namibia; 23.00° S) and (2) the southeast coast, from St Francis Bay (South Africa; 34.00° S) to Beira (Moçambique; 20.00° S). Both coastlines were gridded so the cells would match the spatial resolution of HYCOM (0.08° x 0.08°). Passive particles simulating drifting larvae were released from each coastal cell on a daily basis throughout the spawning season of *M*. *galloprovincialis* (from May to July and from October to January; [71]). The particles were allowed to drift for 30 and 90 days, which are the average and upper end (when metamorphic delay occurs) Pelagic Larval Duration (PLD) known for *Mytilus* spp. [68,72–75]. In these experiments, the geographical position of each particle was determined every two hours using the local bilinear interpolation of HYCOM’s velocity fields. The aggregated trajectories allowed the production of a connectivity matrix between every pair of cells, by determining the number of temporal steps that a particle released from cell i crossed cell j, divided by the number of steps simulated per particle (30 days PLD * 12 steps per day). To account for inter-annual variability, simulations were run individually for each year for a 5-year period (2008 to 2012), and the mean connectivity matrix was calculated by averaging the annual matrices. The null hypotheses of no correlation between the connectivity matrices performed with contrasting PLDs (30 and 90 days) for both west and southeast coasts were tested using Mantel non-parametric test based on 9999 permutations.

Niche modeling analysis, niche divergence tests and dispersal simulations were performed in R [76] using the packages: adehabitat, dismo, gbm, gstat, mda, parallel, raster, SDMTools and vegan.

## Results

**Fig 1:**
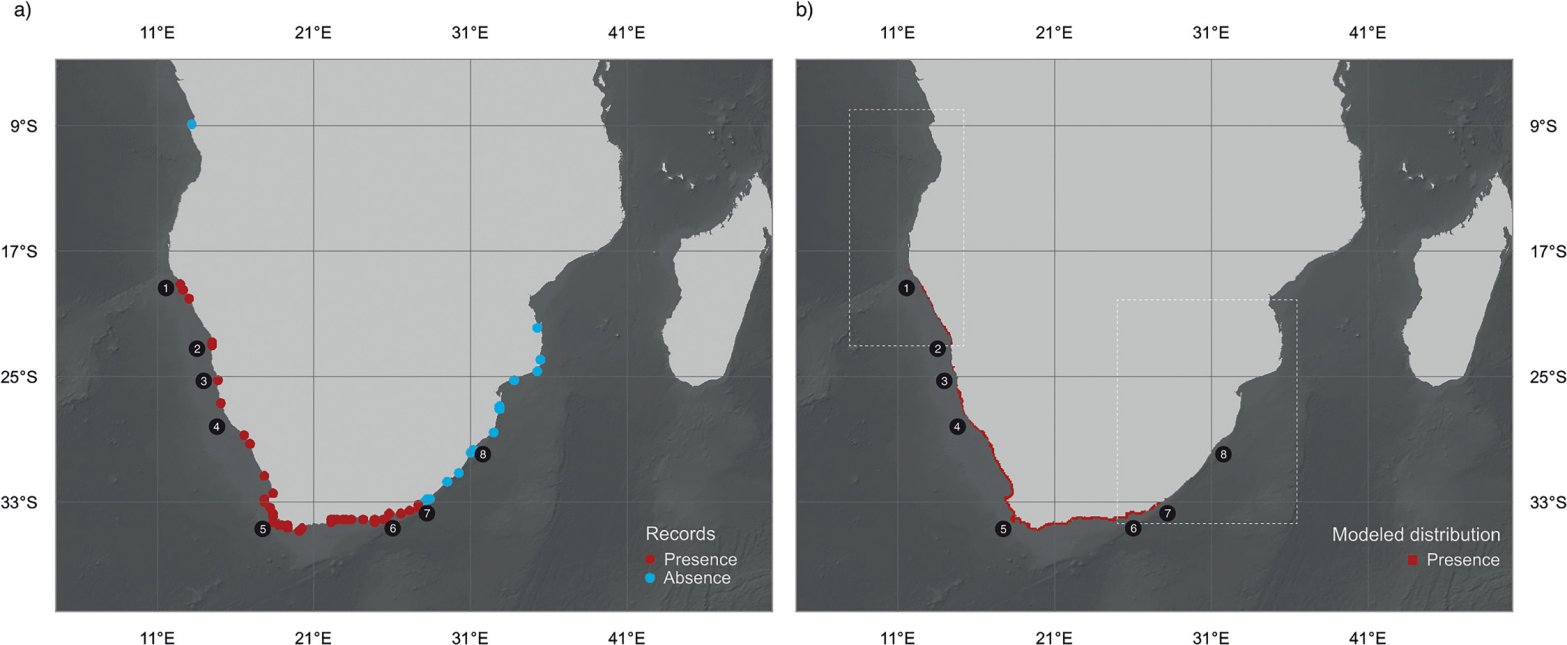
(a) Records of occurrence for *M*. *galloprovincialis* throughout the southern African coast (Red dots for presence and blue dots for absence). (b) Reclassified ensemble showing the occurrence of *M*. *galloprovincialis* for the present (2000–2010). Numbers show Regions Of Interest: ROI 1. Rocky Point, ROI 2. Walvis Bay, ROI 3. Sossusvlei, ROI 4. Elizabeth Bay, ROI 5. Cape Town, ROI 6. Port Elizabeth, ROI 7. East London, ROI 8. Durban. The dashed squares indicate those regions where the Lagrangian Particle Simulations where performed: the west coast, from Capulo to Walvis Bay, and the southeast coast, from St Francis Bay to Beira. Credits for the background of both maps: General Bathymetric Chart of the Oceans (GEBCO).

The field surveys provided 104 georeferenced records for *M*. *galloprovincialis* in southern Africa. For modelling purposes, this dataset was enhanced with 170 additional records from native ranges derived from field surveys in North Africa and Southern Europe, the available literature and an electronic database (Fig 1A; S1 Text).

### Ecological Niche modelling of *M*. *galloprovincialis*

**Fig 2:**
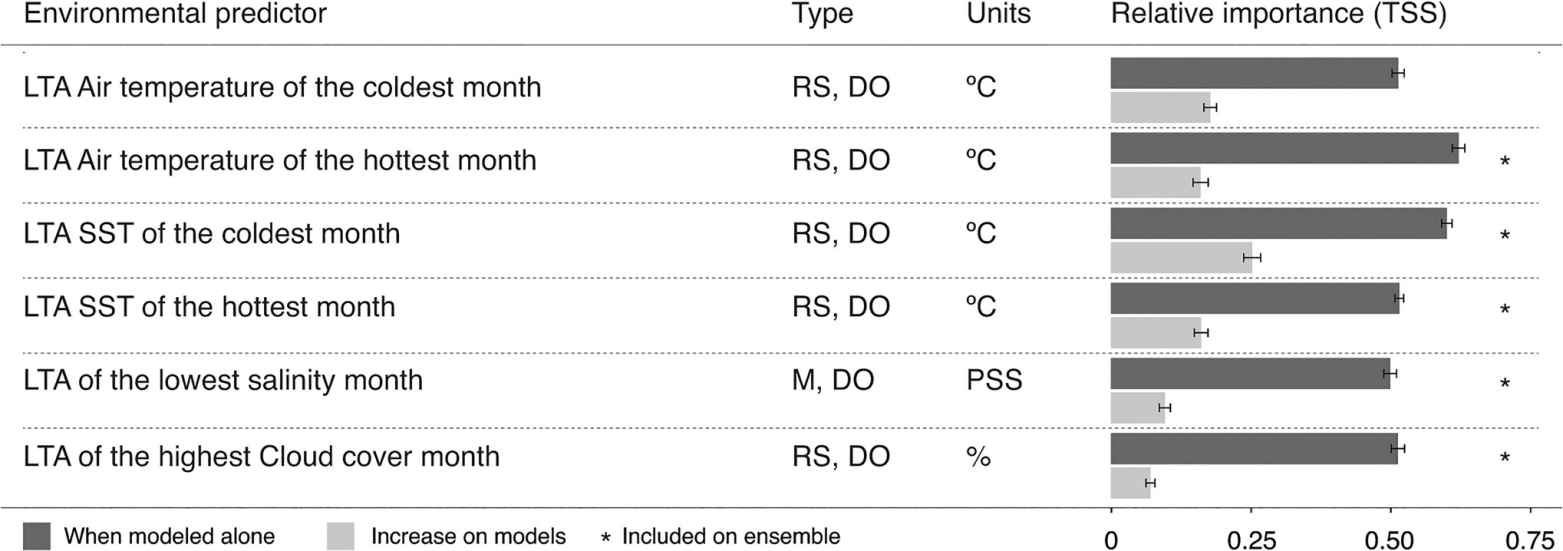
Environmental predictors used in niche modelling as Long Term Averages (LTA), type of data (M—Modelled; RS—Remote sensing; DO—Direct observation), units and relative importance on cross-validation (TSS—True Skill Statistics). Asterisks show the predictors included in the final ensemble.

Measuring the relative importance of each environmental predictor to the response of the models showed that the distribution of *M*. *galloprovincialis* is best explained by the extremes of high air and low sea temperatures (Fig 2). These two predictors retrieved good accuracy scores when modelled alone (TSS > 0.6), while when combined with others, they added gains in TSS from 0.16±0.01 to 0.25±0.01. All other predictors produced null models when used alone, retrieving accuracy scores ranging from 0.48±0.01 (low salinity) to 0.51±0.01 (hottest sea temperatures). Nevertheless, they showed a fair contribution to the models when combined with other predictors, adding accuracy gains from 0.09±0.01 (high cloud cover) up to 0.17±0.01 (coldest air temperatures).

The ensemble produced with the best transferrable models resulted in an excellent description of *M*. *galloprovincialis* distribution (TSS: 0.95), while showing low levels of uncertainty (S1 Fig.; SD ranging from 0 to 0.15). This prediction indicated that this species’ niche is available from Rocky Point in Namibia (ROI 1, Fig 1B) southwards to East London in South Africa (ROI 7). Within this wide region (~2800 km), the predicted niche was not continuous though, showing low suitability scores in parts of Namibia, specifically south of Walvis Bay (ROI 2), in Sossusvlei (ROI 3) and Elizabeth Bay (ROI 4, Fig 1B). Our field surveys substantially corroborated the niche modelling (Fig 1A and 1B), resulting in high specificity and sensitivity scores (1 and 0.97, respectively). While all modelled presences were validated in the field, only one false absence was predicted (at Sylvia Hill, Namibia, near ROI 3).

#### Niche divergence between ranges

**Fig 3:**
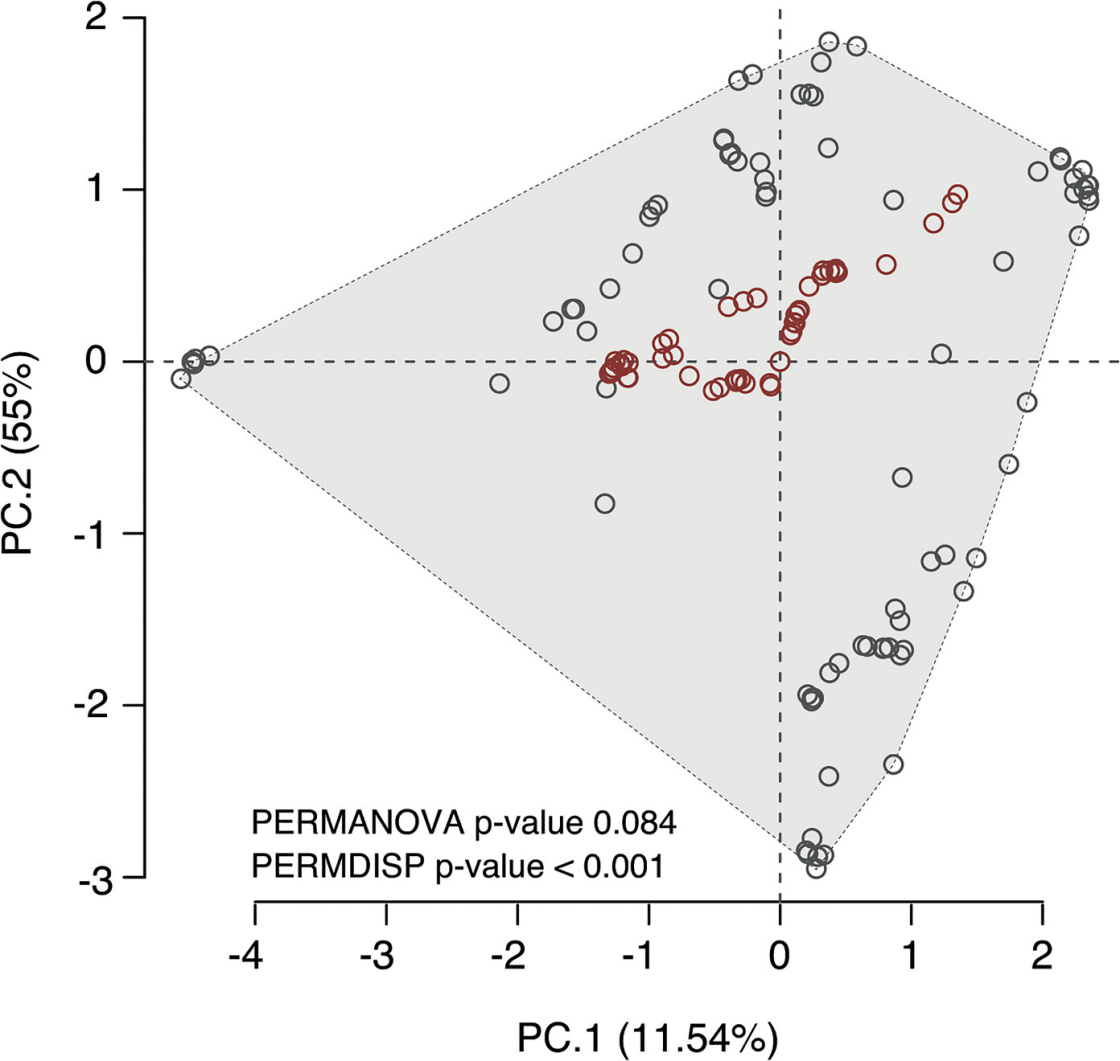
Principal Component Analysis (PCA) of the environmental space used by native (grey circles) and non-native (red circles) occurrences of *M. galloprovincialis*. The grey polygon indicates the overall environmental space of the species. The significance levels of the permutational multivariate ANOVA (PERMANOVA) and permutational analysis of multivariate dispersion (PERMDISP) to test for niche divergence between native and non-native ranges are shown.

The first two PCA components performed to infer niche divergence explained 66.54% of the variability found in the environmental data. This analysis showed that the invasive occurrences use part of the native environmental space (Fig 3). PERMANOVA and PERMDISP further confirmed this, indicating that a niche shift by *M*. *galloprovincialis* during its spread through southern Africa was unlikely (PERMANOVA Pseudo-F: 2.801; p: 0.084) and that the native ecological niche is wider (i.e., more dispersed) than the invaded one (PERMDISP F: 138.1; p: < 0.001).

### *M*. *galloprovincialis* dispersal potential

**Fig 4:**
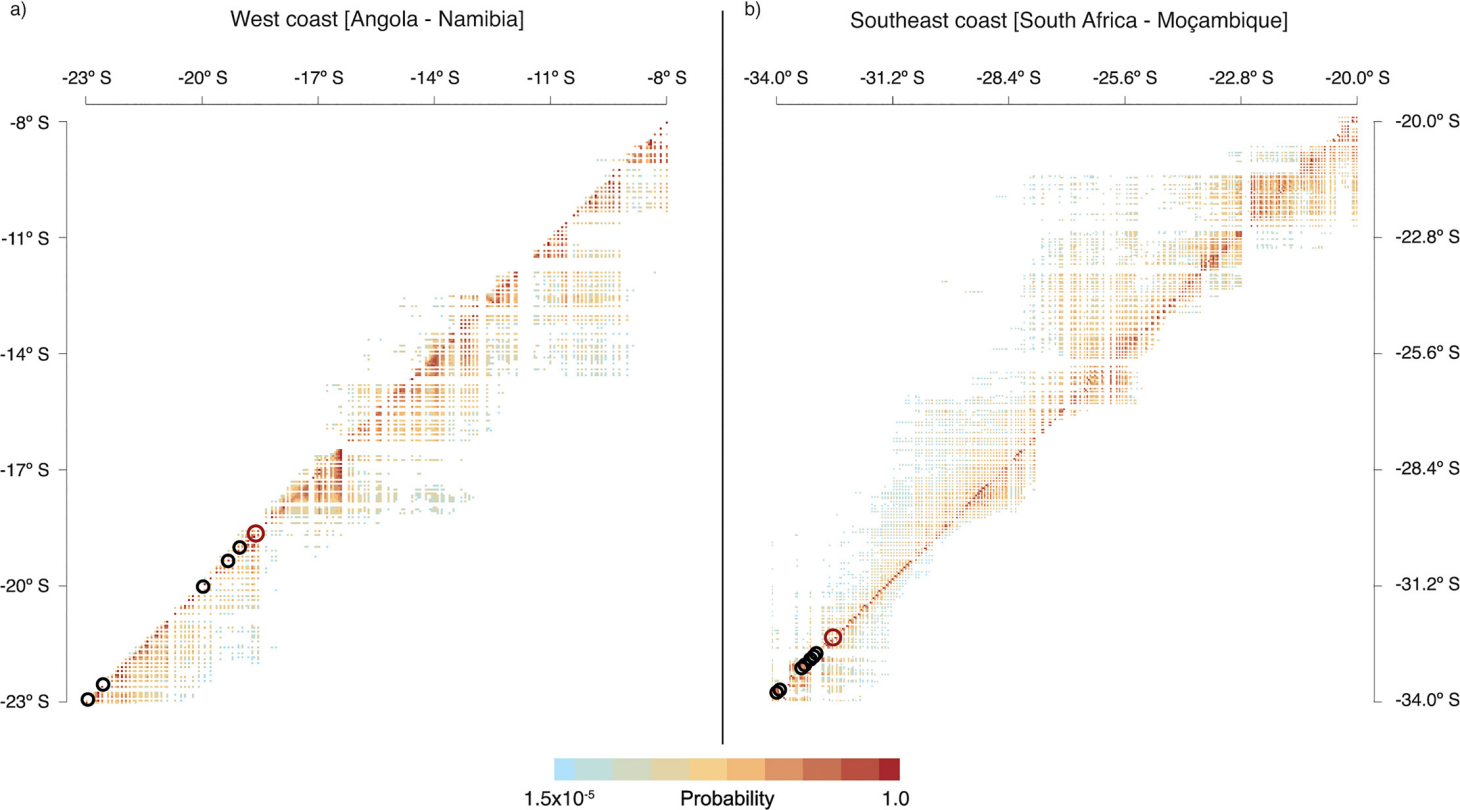
Potential connectivity matrices for the Lagrangian Particle Simulations performed on the (a) west and (b) southeast coasts of southern Africa using a PLD of 30 days. Black circles show locations where *M*. *galloprovincialis* occurs, while the open red circles show where the niche models predicted northern edges.

The particle simulations fed by HYCOM velocity fields over the entire 5-year period allowed the release of 5040 particles per site (3,512,880 tracked particles in total). The aggregated trajectories resulted in different connectivity matrices corresponding to how long the passive particles were allowed to drift (30 or 90 days of PLD) and to where the simulations took place (west or southeast coasts). Regarding the experiments using contrasting PLDs, we verified high resemblances (Fig 4; S2 Fig), no statistical differences and strong correlations between connectivity matrices (west coast: Mantel r = 0.944, p-value < 0.001; southeast coast: Mantel r = 0.927, p-value < 0.001). Therefore, we centre further analysis and assumptions on the matrices produced for the west and southeast coasts using 30 days PLD, which is the more likely scenario for pelagic states of *M*. *galloprovincialis*. These matrices showed a high probability for larval retention (matrix’ diagonals), although their general trends were found to be contrasting. The particles tracked on the west coast (Fig 4A) displayed a marked northward trend, with an exception from Benguela (12.50° S) to Tombua (16.50° S), where particles also moved southward, though for shorter distances. On the southeast coast of Africa (Fig 4B) the particles moved mostly along southward trajectories. In this region, with the exception of a few oceanographic discontinuities, particles were also found to move northward, though for shorter distances when compared to the general trends for this region.

The inferred trajectories indicate that particles released from Rocky Point and Maui Bay (northern locations where *M*. *galloprovincialis* occurs on the west coast) have little probability of moving northward (probabilities < 0.05) due to a strong oceanographic discontinuity found at Cape Frio (18.50° S; close to ROI 1, Fig 1). On the southeast coast, particles released from Kidd’s Beach and Kayser’s Beach (close to the most easterly locations for *M*. *galloprovincialis* on the southeast coast) may move further to the northeast, up to the shores located at 32.60° S (probabilities > 0.05, Fig 4). In a year-to-year stepping-stone scenario, particles released from these two locations could move further to the northeast, although they never crossed the oceanographic discontinuities found at Durban (29.80° S) or near Kosi Bay (27.00° S).

## Discussion

### The distribution of *M*. *galloprovincialis* in southern Africa

The contribution of each environmental predictor to the models was corroborated by previous studies focused on the physiology of *M*. *galloprovincialis*, which showed that extreme air and sea temperatures greatly influence its growth and survival [26,49–51]. The strong effects of temperature were further substantiated by the distributional limits inferred by the models, which coincide with steep transitions between cool temperate and warmer subtropical climates. The first is located in the proximity of the Angola-Benguela Front (16^o^S; [77]). This region lies at the convergence of the south-flowing Angola Current and the north-flowing cold water of the Benguela upwelling system and corresponds to the northern boundary of coastal upwelling off the Namibian coast [78–79]. The second distributional limit for *M*. *galloprovincialis* occurs on the South African southeast coast (32^o^S). There, the continental shelf gradually widens, deflecting the warm Agulhas current, which flows to the south-west following the continental break, away from the coast, thus reducing sea surface temperatures significantly [80]. Because warm waters tend to have less nutrients than cold waters [81], the distributional limits of *M*. *galloprovincialis* may also be affected by unfavourable trophic conditions beyond these boundaries, in addition to prohibitive thermal conditions. This effect could be particularly evident during the austral summer, when upwelling conditions there are minimal [82], reducing the availability of plankton throughout southern Africa [83].

The additional environmental predictors used in our framework also produced fair contributions to the accuracy of models. Several authors have shown that distribution patterns of several *Mytilus* spp. and their hybrids correlate with salinity gradients (e.g., [84–85]). The influence of salinity is further supported by laboratory experimental evidence showing that it affects the physiology (i.e. heart rate) and behaviour (i.e. valve closure) of *Mytilidae* [49]. Furthermore, low cloud cover may interact significantly with other environmental variables affecting the thermal stress experienced by intertidal organisms [86] by reducing the importance of solar radiation and therefore lowering mussels’ body temperatures significantly [87]. In fact, this is a prominent feature in the study region, particularly throughout the Namibian shores, where the interaction between cold Benguela water and air temperature saturates the low altitude climate with moisture from the sea.

The total agreement found between modelled occurrences and the realized invasive distribution (sensitivity = 1) allowed us to deduce that *M*. *galloprovincialis* has already occupied the entire ~2800 km of suitable habitat in southern Africa since its arrival in the late 1970s. Soon after its arrival in South Africa, *M*. *galloprovincialis* spread rapidly to the north at an average speed of 115 km yr^–1^ and to the south at about 25 km yr^–1^ [26]. In the last 20 years, its expansion on the south coast has essentially ceased, and it has previously been suggested that it might have now reached stability at its eastern limit [88]. Our study is the first to explicitly assess the distribution of this invader and to evaluate environmental determinants of its distributional limits. Furthermore, our results suggest that such rapid spread was not based on adaptation to novel environmental conditions, as niche adaptation was not demonstrated; the invasive populations inhabiting southern Africa shores currently use a narrower part of the native environmental space. Rather the spread of *M*. *galloprovincialis* must have relied on very efficient dispersal of planktotrophic larvae and the better physiological performance of the species compared to native mussels (e.g., [37]).

While our distribution models only considered climate predictors, biotic conditions may also play an important role in the success of a potential invasion. An invasive species must interact with the native species and such interactions can be fundamental to invasion dynamics. On the west coast, the spread of *M*. *galloprovincialis* was likely facilitated by its superior competitive characteristics compared to the native mussels *Aulcaomya ater* and *Choromytilus meridionalis*, and it rapidly displaced them to become the dominant intertidal mussel [27]. In contrast, on the south coast, *M*. *galloprovincialis* interacts with a different native species, *Perna perna*, and the two show partial habitat segregation [26], largely based on their differing competitive abilities across the steep environmental gradient of the intertidal [89].

### Dispersal of larvae beyond present limits

Our dispersal simulations are in agreement with the directionality of two main oceanographic features characterizing the western and southeastern coasts, suggesting that the methodology implemented here can provide a reasonable estimate of particle transport across main current flows (as previously shown by [17]). On the west coast, particles followed the predominant northward flow of the Benguela current and, to a lesser degree, the flow of the Angola Current (from 12.50° to 16.50° S; [68]). Both currents deflect westwards, bringing intense upwelling and transporting particles offshore [77,90]. This effect was particularly evident at Cape Frio, at the northern boundary of the Benguela front, where we inferred a permanent dispersal barrier that limits the transport of *M*. *galloprovincialis* larvae to latitudes further north [90–91], even when considering extreme pelagic durations of up to 90 days (S2 Fig).

All suitable habitat has been occupied by *M*. *galloprovincialis* along the west coastline as far as the northern distributional limit. This is probably a result of two main factors: a) the species’ high reproductive output and capacity to colonize new areas [26,92–93] and b) the strong northward flow of the Benguela Current, which would have favoured an initial, strongly unidirectional spread of *M*. *galloprovincialis* along this coast [34]. Although these factors would promote rapid spread of the species, environmental conditions are unsuitable beyond the present northern limit; probably inhibiting any settlement of larvae passing through the physical barrier at Cape Frio.

On the southeast coast, the main trend observed in the simulations was caused by the prevailing fast-flowing Agulhas Current, which pushed most particles southwestwards. This is in agreement with previous studies that employed nearshore drogues. Drifters released off the south coast were caught by the Agulhas Current and moved primarily offshore and towards the southwest [94]. However, in contrast to our findings, there was remarkably little overlap between the trajectories of drifters released off the south coast and those released on the east coast, suggesting that ocean dynamics may limit larval dispersal between the two regions. Instead, our simulations supported some potential eastward movement of drifting larvae, beyond the present limits of *M*. *galloprovincialis* along this coast (East London, South Africa, Fig 1B). This may be associated with wind-driven surface currents as they have been previously demonstrated to be an important element of mussel larvae transport along the southeast coast [68]. Inshore counter-currents that lie closer to the coast (< 2–3 km) than the release points of drogues may also allow larvae to disperse to the northeast against the general trend imposed by the strong Agulhas Current. In fact, larvae of *M*. *galloprovincialis* (identified following [26]) have been recently collected using a plankton pump near Mazeppa Bay (~85 km east of East London, 32° 29'S 28° 39'E) at very low densities (1 *M*. *galloprovincialis* larvae per ~900 L of seawater) [95]. Thus, occasional dispersal as much as 150 km beyond the present distributional limit does occur, although our simulations indicate that larvae are unlikely to cross strong environmental discontinuities further to the northeast at Durban and Kosi Bay.

As larvae can pass beyond the present established range, the complete absence of adults/recruits beyond established ranges (as inferred by field surveys and niche models) shows that environmental conditions are major drivers of local selection. Both northern ranges of *M*. *galloprovincialis* currently coincide with a strong environmental transition between the warm sea temperate (15–22°C) and subtropical bioregions (22–27°C) [96]. These boundaries characterizing the species’ one-dimensional distribution along the coastline presumably indicate a permanent physiological barrier, beyond which environmental conditions fall outside this species’ tolerances. Such steep transitions defined by climate are also phylogenetic breaks for two other coastal species, the native mussel *P*. *perna* and the estuarine mudprawn *Upogebia africana* [97]. It has been suggested that as climate change raises temperatures in the near future, the niche of *M*. *galloprovincialis* may become more common throughout the globe, thereby favouring the further spread of this species [51]. While this may be true for other warm-temperate regions, our results suggest that the distribution of *M*. *galloprovincialis* may become more constrained in southern Africa due to the advance of the subtropical isotherms to higher latitudes [98], where this species is already present. Furthermore, because upwelling systems of the world are predicted to become more intense in the face of climatic changes [99], the current oceanographic discontinuities inferred in our study may become stronger and more permanent, further limiting the distribution of this species.

We draw our main conclusions that the limiting of *M*. *galloprovincialis* post-invasion spread is strictly through ocean currents. While our simulations are based on the actual oceanographic patterns of southern Africa, which are presumably the main drivers of the spread of this species after its earlier colonisation stages, we cannot rule out dispersal thorough subsequent, human-mediated introductions. It is known that the maritime network of cargo ships plays a crucial role, with seawater discharges from ballast tanks and hull fouling seen as major vectors for biological invasions between ports worldwide [100–102]. These processes may have assisted the spread of *M*. *galloprovincialis* in our study region, particularly on the southern shores, where major ports structure a local network of trading that has intensified in the last decades between Cape Town, Port Elizabeth, East London and Durban (ROI 5–8, Fig 1) [102–103]. Nowadays, since all suitable habitats are fully occupied (as shown by our results), these nodes may be contributing to a mere homogenization effect among populations of *M*. *galloprovincialis* (e.g., [104]) and not to promote the further spread of the species throughout southern African shores.

Our main findings broaden our knowledge about the processes driving the spread of *M*. *galloprovincialis*, but may be extended to the global invasion dynamics of marine species relying on pelagic larvae as the primary dispersal vector. A recent assessment of invasive marine species in our study region revealed 86 introduced and 39 cryptogenic species, of which 13 are bivalves [105]. Among these, *Semimytilus algosus* has recently spread along the west coast of South Africa [106] using an identical dispersal strategy as *M*. *galloprovincialis* ([107] and references therein). The distributional range of *S*. *algosus* now extends for approximately 500km and it occupies lower portions of the mussel zone below *M*. *galloprovincialis* [106]. Given the high pelagic larvae duration and strong competitive abilities of both species, it is likely that environmental conditions alone play the crucial role limiting their spread from the initial sources of introduction. If ocean currents favour dispersal of other bivalve species with the same potential for spreading and competition, invasions may cover broad regions (in the orders of 1000s km) in just a few decades, reshaping the population structure of native intertidal communities, like those already impacted in southern Africa by *M*. *galloprovincialis* and *S*. *algosus* [105].

## Supporting Information

S1 Fig Maps showing (a) the probability of occurrence of *M*. *galloprovincialis* in southern Africa derived from the ensemble of the best transferrable niche models and (b) the uncertainty of the ensemble given by the Standard Deviation (SD). Credits for the background of both maps: General Bathymetric Chart of the Oceans (GEBCO).
(TIF)

S2 Fig Potential connectivity matrices for the Lagrangian Particle Simulations performed on the (a) west and (b) southeast coasts of southern Africa using a PLD of 90 days. Black circles show locations where *M*. *galloprovincialis* occurs, while the open red circles show where the niche models predicted northern edges.
(TIF)

S1 Text Records used to build the niche models with information on year of collection, source of data, longitude (Lon), latitude, (Lat), Occurrence (1 for presence and 0 for absence), range (native or non-native) and reference (if applicable). (CSV)
