# Supplementary figures and images for: Oceanographic Conditions Limit the Spread of a Marine Invader along Southern African Shores
Source: PLoS One. 2015 Jun 26;10(6):e0128124. doi: 10.1371/journal.pone.0128124 (PMC4482700; doi:10.1371/journal.pone.0128124)

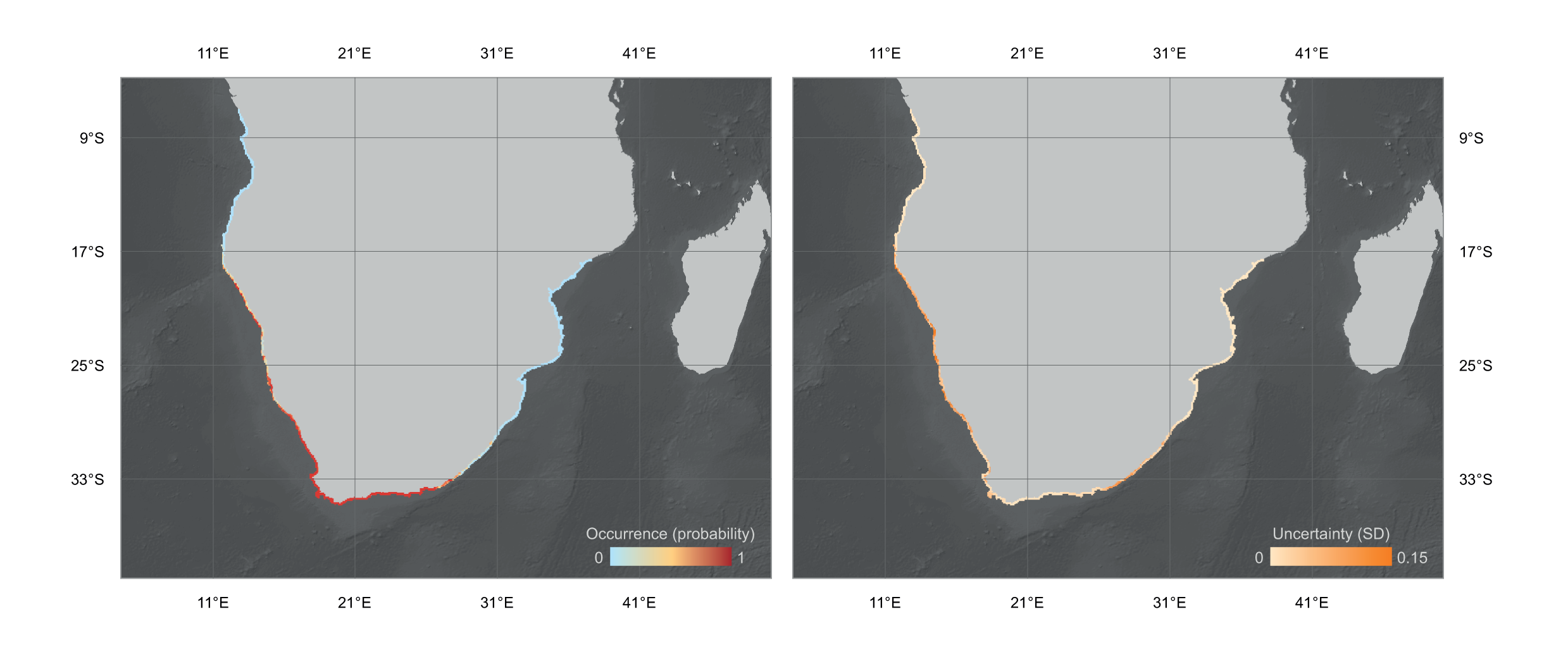

Supplement: S1 Fig — Credits for the background of both maps: General Bathymetric Chart of the Oceans (GEBCO). (TIF) [file pone.0128124.s001.tif]

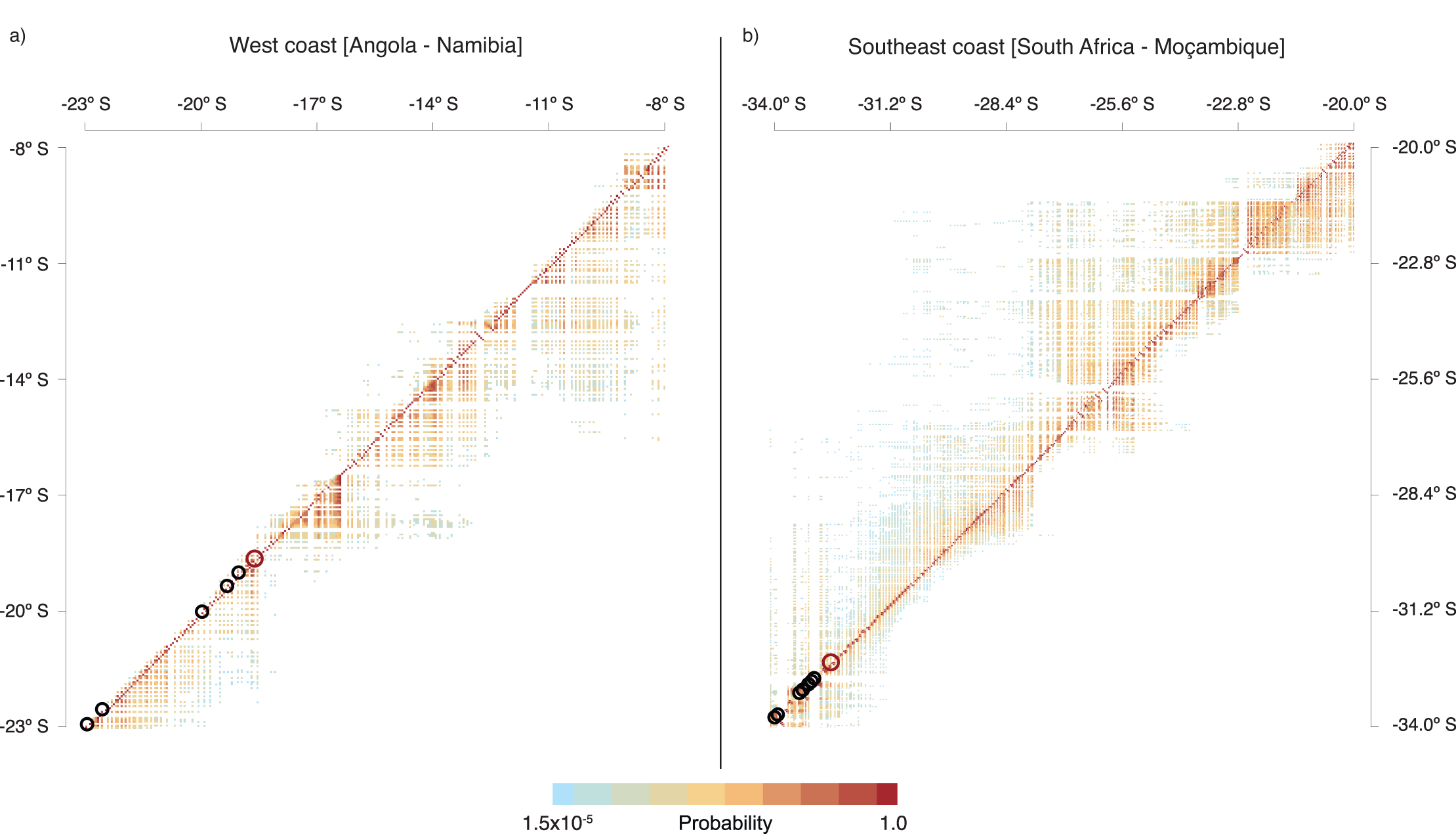

Supplement: S2 Fig — Black circles show locations where M. galloprovincialis occurs, while the open red circles show where the niche models predicted northern edges. (TIF) [file pone.0128124.s002.tif]
